# Serum epidermal growth factor receptor and HER2 expression in primary and metastatic breast cancer patients

**DOI:** 10.1186/bcr1788

**Published:** 2007-11-01

**Authors:** Kristjan S Asgeirsson, Amit Agrawal, Claire Allen, Anthony Hitch, Ian O Ellis, Caroline Chapman, Kwok L Cheung, John FR Robertson

**Affiliations:** 1 Division of Breast Surgery, University of Nottingham, Nottingham City Hospital, Hucknall Road, Nottingham NG5 1PB, UK; 2 Division of Histopathology, University of Nottingham, Nottingham City Hospital, Hucknall Road, Nottingham NG5 1PB, UK; 3 Department of Clinical Chemistry, Nottingham City Hospital, Hucknall Road, Nottingham, NG5 1PB, UK

## Abstract

**Background:**

Breast tissue expression of the *ERBB *proto-oncogene family has been extensively studied. It was recently shown that expression of epidermal growth factor receptor (EGFR; c-erbB-1) and epidermal growth factor receptor (HER)2 (c-erbB-2) can be detected in the serum of breast cancer patients. The clinical relevance of this has not been fully established.

**Methods:**

EGFR and HER2 immunoassays were conducted in blood from 57 patients in whom paired serum samples were available (from the times of primary and metastatic diagnoses), from 96 primary breast cancer patients and from 49 normal individuals. Of the 57 patients with paired serum samples, paired tissue samples for HER2 expression were available for eight.

**Results:**

Serum levels of EGFR serum levels were significantly higher in normal individuals (median 75.3 ng/ml, range 43.2 to 114.2 ng/ml) than in patients with primary breast cancer (median 59.3 ng/ml, range 21.3 to 94.1 ng/ml; *P *< 0.001). In the paired serum samples, EGFR levels decreased significantly between the time of primary diagnosis (median 56.3 ng/ml, range 29.1 to 142.7 ng/ml) and metastatic diagnosis (median 30.9 ng/ml, range 10.9 to 106.4 ng/ml; *P *< 0.001). In six (11%) a change occurred from over-expression (>78 ng/ml) to normal expression. In contrast, no significant difference was seen in HER2 serum levels in normal individuals (median 12.2 ng/ml, range 7.8 to 20.9 ng/ml) and primary breast cancer patients (median 12.5 ng/ml, range 6.9 to 122.2 ng/ml; *P *= 0.511). However, in the paired serum samples, HER2 levels increased significantly between the time of primary (median 12.2 ng/ml, range 5.7 to 85.0 ng/ml) and metastasis (median 17.7 ng/ml, range 6.3 to 3,337.4 ng/ml; *P *< 0.001). HER2 over-expression (>15 ng/ml) was observed in 16 out of 57 patients (28%) at primary breast cancer diagnosis and in 31 out of 57 (54%) at metastasis. In 18 patients (32%) HER2 expression changed from normal to over-expression.

**Conclusion:**

Decisions regarding the use of targeted therapies in the metastatic setting are often based on the oncogene expression of the primary tumour. Our results suggest that serum oncogene assessments may be complementary to this and could potentially widen the indications for these beneficial therapies.

## Introduction

The erbB (human epidermal growth factor receptor [HER]/neu 1 to 4) receptors are a family of four known receptors localized on the cell membrane of most tumours, including breast cancer. These receptors are activated by various ligands that trigger a network of signalling pathways, leading to increased uncontrolled growth of cancer cells and unfavourable prognosis. The over-expression of EGFR (erbB-1 receptor) is seen in 20% to 81% of breast tumours, whereas HER2 receptor over-expression is seen in approximately 20% [1-4].

The extracellular binding region of these two receptors for EGFR and HER2 are proteolytically released from the cell surface upon receptor activation and can be detected in patients' serum. The relevance of the serum expression of these proteins has been the subject of a number of studies [5-7]. Early studies conducted by Mansour and coworkers [8] showed that serum HER2 levels were significantly higher in patients with breast cancer than in patients with benign breast diseases and in normal control individuals. Also, in this group of breast cancer patients, serum HER2 positivity was a significant prognostic factor [8]. Others showed that serum HER2 positivity was significantly more common in patients with recurrent breast cancer than in those with primary breast cancer, and that there was a good association between HER2 tissue expression and serum HER2 levels [9]. Measuring this protein in the serum could provide an easier and more accessible method of diagnosing patients who may benefit from targeted therapies (trastuzumab) as compared with assessment of tissue expression. Also, it has opened the possibility of measuring the 'real-time' expression of this oncogene, which may be useful in assessing treatment effects [10,11].

The potential role for serum EGFR assessments in breast cancer is not quite as clear, however. Although raised pretreatment levels have been shown to be associated with decreased progression-free and overall survival in metastatic breast cancer patients [6,12], several studies have shown that patients with some solid epithelial cancers in fact exhibit decreased levels of serum EGFR compared with healthy control individuals [6,13]. The biological background and the clinical relevance of these observations are not yet known.

Presently anti-EGFR/HER2 therapy for recurrent/metastatic breast cancer is based on immunohistochemistry and fluorescence *in situ *hybridization of the primary breast cancer tissue [14]. Still, the value of the serum expression of these markers in predicting response to such therapies remains largely unclear. In the present study, we analyzed serum expression of EGFR and HER2 in paired samples (at time of primary diagnosis and at metastasis) and we aimed to correlate this with the tissue expression. Furthermore, we analyzed the serum expression of these markers in an unselected group of healthy individuals and consecutive breast cancer patients diagnosed in our unit. Finally, the association of these with tumour markers carcinoembryonic antigen (CEA) and cancer antigen (CA)15.3 was determined.

## Materials and methods

### Patients

Between October 1992 and October 2004, 57 patients were identified from whom paired blood samples were available for analysis (both at diagnosis of primary breast cancer [PBC] and at metastasis). Blood samples were collected from patients attending breast clinics who gave written, informed consent under a study protocol approved by the institutional ethics committee. Blood specimens were allowed to stand before being centrifugated at 1,250 *g *for 5 minutes, serum removed and stored at -20°C.

Furthermore, preoperative serum samples were collected from 96 PBC patients and 49 healthy individuals attending the National Health Service Breast Screening Programme and had a normal mammography or were attending a symptomatic breast clinic and discharged with no detected abnormality following triple assessment (age range 19 to 71 years, mean age 54 years). All PBC and normal samples from healthy individuals were collected during a 12-month period in 2004. Of the 57 patients with paired blood samples, paired tissue samples (primary and metastasis) were also available from 8.

Clinical data were collected from the unit's database, which was prospectively updated (following each clinic consultation). Any change in a patient's treatment or progress was entered into our database.

### Methods

Serum EGFR and HER2 estimation was done using Bayer microtitre Immunoassays (ADVIA Centaur^® ^Immunoassay system, Bayer Healthcare Diagnostics Divisions, Bayer House, Strawberry Hill, Newbury BERK, RG14 1JA, UK). The normal range for EGFR was 45 to 78 ng/ml and for HER2 it was under 15 ng/ml (based on the mean value of healthy individual ± 2 standard deviations). Tissue HER2 expression was estimated using the HercepTest kit protocol (Dako Ltd, Denmark House, Angel Drove, Ely, Cambridgeshire, CB7 4ET, UK). The immunohistochemical expression was quantified as 0, 1+, 2+, or 3+. HER2 positivity was defined as 3+. Tissue EGFR immunohistochemistry was performed with mouse monoclonal antibodies against EGFR in accordance with the protocol provided by Novocastra Ltd and the expression was scored according to the percentage of cells exhibiting either membrane or cytoplasmic staining (Novocastra Laboratories Ltd, Newcastle upon Tyne, NE12 8EW, UK). The cut-off for positive expression was defined as 10% or more of cells stained. Serum CA15.3 and CEA estimation was done using Automated Immunoassay on the Bayer Centaur Immunoassay analyzer. The normal range for CA15.3 was 0 to 35 kU/l and for CEA it was 0 to 5 kU/l.

Tumour grade was assessed using the Nottingham method [15]. Lymph node stage was determined from the results of axillary node sampling or dissection. For patients diagnosed with primary breast cancer before 1995, oestrogen receptor (ER) activity was measured using the standard radioligand binding assay on tissue cytosol samples, with the threshold for designation of ER positivity being 10 fmol/mg of protein. Since then, ER status has been determined using immunocytochemical methods. An H-score above 50 has been used to identify ER positive tumours for adjuvant endocrine therapy.

### Statistical analysis

Statistical analysis was performed with the SPSS software package (version 12.0 for Windows; SPSS Inc., Burlingame, CA, USA). Because the distribution of serum EGFR and serum HER2 levels was nonparametric, the Mann-Whitney test was used to compare median values. χ^2 ^analysis and Fisher's exact were used to compare numbers in each group. A *P *value of less than 0.05 was considered statistically significant.

## Results

### EGFR and HER2 serum expression in healthy individuals versus breast cancer patients

Healthy individuals had significantly higher serum EGFR levels (median 75.3 ng/ml, range 43.2 to 114.2 ng/ml) than did the PBC patients (median 59.3 ng/ml, range 21.3 to 94.1 ng/ml; U = 3,599.0, *P *< 0.001)). For serum HER2, no significant difference was observed between the healthy individuals and PBC patients (median 12.2 ng/ml, range 7.8 to 20.9 ng/ml and median 12.5 ng/ml, range 6.9 to 122.2 ng/ml, respectively; U = 2,383.5, *P *= 0.511). Ten out of 96 (10.4%) PBC patients had serum EGFR levels above the reference range (45 to 78 ng/ml), as compared with 20 out of 49 (40.8%) healthy individuals. For serum HER2 (reference range < 15 ng/ml), the respective numbers were 22 out of 96 (22.9%) and 10 out of 49 (20.4%).

### Tumour characteristics of patients for whom paired blood samples were available

The demographics of the 57 patients and their tumour characteristics are shown in Table 1. The median interval between primary and metastatic diagnosis was 23 months (range 5 to 132 months).

**Table 1:** Tumour characteristics where paired serum available

Characteristic		Value
Tumour size	T1	21 (36.8)
	T2	30 (52.6)
	T3	5 (8.8)
	Not known	1 (1.8)
Grade	1	2 (3.5)
	2	14 (24.6)
	3	35 (61.4)
	Not known	6 (10.5)
Lymph node status	None	22 (38.6)
	1 to 3	22 (38.6)
	>3	10 (17.5)
	Not known	3 (5.3)
NPI	≤3.4	5 (8.8)
	3.5 to 5.4	23 (40.4)
	>5.4	19 (38.3)
	Not known	10 (17.5)
ER status	Negative	22 (38.6)
Age (years)		53 (30–86)

Paired serum samples were available for 57 patients. Values expressed as *n *(%) apart from age, which is expressed as median (range). ER, oestrogen receptor; NPI, Nottingham Prognostic Index.

#### EGFR and HER2 serum levels at time of primary and subsequent metastatic diagnosis

**Figure 1:**
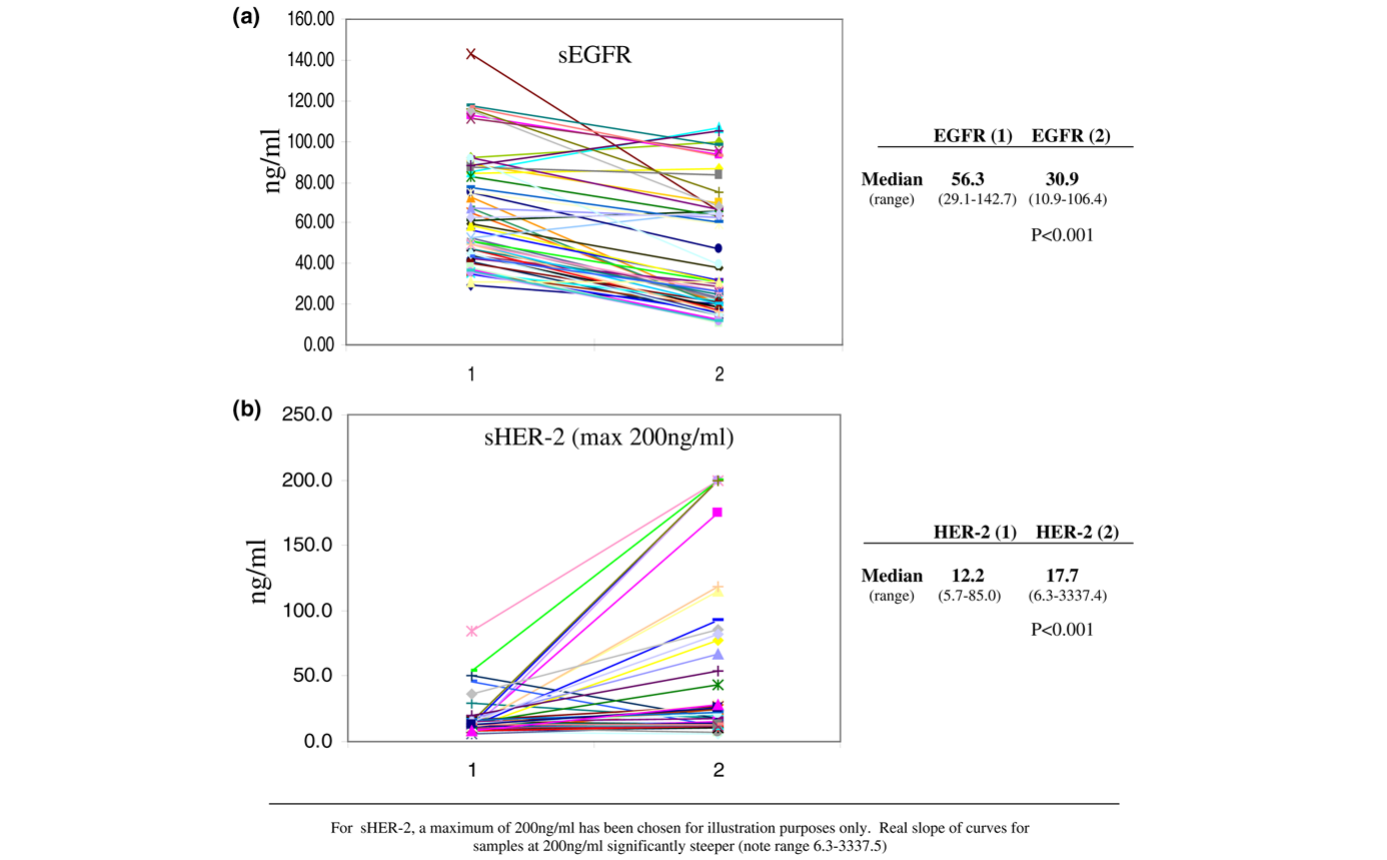
EGFR and HER-2 serum levels. **(a) **Epidermal growth factor receptor (EGFR) and **(b) **human epidermal growth factor receptor (HER)2 serum levels at time of (1) primary and (2) metastatic diagnosis. sEGFR, serum EGFR; sHER2, serum HER2.

EGFR levels were significantly higher at the time of primary diagnosis (median 56.3 ng/ml, range 29.1 to 142.7 ng/ml) than at the time of metastatic diagnosis (median 30.9 ng/ml, range 10.9 to 106.4 ng/ml; U = 2168.0, *P *< 0.001). Eighteen of the 57 patients (31.6%) had elevated serum EGFR levels at primary diagnosis. In six (11%) a change occurred from over-expression (>78 ng/ml) to normal expression (45 to 78 ng/ml). No patient changed from normal expression at primary diagnosis to over-expression at metastasis (Figure 1).

The converse was true for HER2; the levels were significantly higher at metastasis (median 12.2 ng/ml, range 5.7 to 85.0 ng/ml) than at presentation of PBC (median 17.7 ng/ml, range 6.3 to 3,337.4 ng/ml; *P *< 0.001). Sixteen of the 57 patients (28.2%) had elevated serum HER2 at the time of primary diagnosis. In 18 (32%) a change occurred from normal expression (<15 ng/ml) to over-expression (>15 ng/ml). In five (8.8%) a change occurred in the opposite direction, whereas in 34 (59.6%) levels remained in the same group of expression (normal or overexpression) between event periods (Figure 1).

At diagnosis of metastasis, serum EGFR levels were elevated (>78 ng/ml) in 12 patients (21.1%), whereas serum HER2 was elevated (>15 ng/ml) in 31 (54.4%). In seven patients (12.3%) both markers were elevated.

#### Association between change in serum HER-2 and serum EGFR expression and adjuvant hormone therapy

Of 57 patients 35 were ER positive, and notes on assessment of treatment were available for 28. Of these, 16 patients were not receiving adjuvant hormone treatment; of remaining 12 were receiving tamoxifen (*n *= 10), goserelin and tamoxifen (*n *= 1), or anastrozole (*n *= 1). Table 2 shows the association between receiving or not receiving hormone treatment at the time of metastatic diagnosis, and whether a change occurred in serum HER2 or serum EGFR. Adjuvant hormone treatment was significantly associated with no change (reduction) in serum EGFR levels between primary diagnosis and metastasis (*P *= 0.039). No such treatment effect was seen with regard to serum HER2 levels.

**Table 2:** The association between adjuvant hormone treatment and change in serum EGFR and serum HER2 levels

		Change in serum EGFR		Change in serum HER2	
		
		No	Yes	No	Yes
On adjuvant treatment	No	8	8	8	8
	Yes	11	1	8	4
			*P *= 0.039		*P *= 0.459

EGFR, epidermal growth factor receptor; HER, human epidermal growth factor receptor.

#### HER2 and EGFR expression in tissue samples from primary and subsequent metastatic disease

Of the 57 patients from whom paired serum samples were available (primary and metastatic), in only eight was metastatic breast tissue available for analysis. The tissue obtained from these samples were as follows: three skin recurrences, four regional recurrences and one in-breast recurrence. All five patients with regional recurrences and in-breast recurrences were also diagnosed with distant metastases at the same time. Of these eight recurrences in total, seven had no HER2 expression at the time of primary diagnosis and one exhibited over-expression (3+) on immunohistochemistry. Of these seven with no HER2 tissue expression at primary diagnosis, none exhibited a change in tissue expression at metastasis. In only one of these seven was a change in serum HER2 expression from normal to over-expression observed between events. The remaining patient with tissue over-expression at both events exhibited a change in serum HER2 expression between events (from normal to over-expression).

Only five tissue samples were available for EGFR tissue expression analysis (one regional recurrence, three skin recurrences and one in-breast recurrence). When membrane staining was assessed, one of these five samples changed from negative expression to positive (>10% of cells expressing EGFR). Cytoplasmic staining was unchanged in all five samples (all five exhibited positive expression both at primary diagnosis and metastasis), although an increase in the percentage of cells expressing EGFR was seen in two (of which one also exhibited a change in membrane expression). In one of these the cell expression changed from 40% to 60% and in the other from 30% to 80%. Neither one of these samples exhibited a difference in serum EGFR expression between primary diagnosis and metastasis, although two of the remaining three exhibited a decrease in serum EGFR expression (from over-expression to normal levels of expression).

#### Association between serum HER2 and EGFR expression and tumour marker expression at metastasis

Table 3 shows the serum levels of oncogene and tumour marker expression at time of metastatic diagnosis. All four markers were available for 50 patients. In 52% of the patients (26/50) either tumour marker CA15.3 or CEA was elevated. In 64% (32/50) either HER2 or EGFR was elevated, and in 78% (39/50) one of these four markers was elevated. There was a significant association between a raised HER2 serum expression and raised serum levels of the tumour markers CA15.3 (*P *= 0.019) and CEA (*P *= 0.005; Table 4). Such an association was not observed between EGFR serum expression and these tumour markers (Table 4).

**Table 3:** Serum oncogene/tumour marker over-expression

Status at metastasis	NME	EGFR	HER2	EGFR or HER2	CA15.3	CEA	CA15.3 or CEA	EGFR or HER2, or CA15.3 or CEA
Number (out of 50)	11	9	29	32	26	11	26	39
%	22	18	58	64	52	22	52	78

CA, cancer antigen; CEA, carcinoembryonic antigen; EGFR, epidermal growth factor receptor; HER, human epidermal growth factor receptor; NME, no marker elevation.

**Table 4:** Association between HER2 and EGFR expression and tumour marker expression at metastasis

	CA15.3 expression		CEA expression	
	
	Normal	Over-expressed	Normal	Over-expressed
HER2 expression				
Normal	12	5	17	0
Over-expressed	10	19	19	10
		*P *= 0.019		*P *= 0.005
EGFR expression				
Normal	17	20	29	8
Over-expressed	5	4	7	2
		*P *= 0.718		*P *= 0.392

CA, cancer antigen; CEA, carcinoembryonic antigen; EGFR, epidermal growth factor receptor; HER, human epidermal growth factor receptor.

## Discussion

In this study, we have shown that both sEGFR and sHER2 levels change significantly between the times of primary and metastatic diagnosis, albeit in opposite directions. We have also shown that serum EGFR levels are significantly higher in healthy individuals than in PBC patients. Finally, we found a rise in serum HER2 expression to be associated with raised tumour marker levels measured at metastasis.

Furthemore, our results show that a change in sHER2 expression is a common feature observed between the time of primary and metastatic diagnosis. In our study, serum HER2 expression changed in 32% of patients. There are several possible explanations for this. It is increasingly being recognized that individual breast cancers are composed of biologically and genetically different clones of cells [16,17]. The capacity to form metastases may be confined to a particular cellular clone, which may during the progression of the disease result in heterogeneity between the primary and metastases. Another possibility, especially in light of the association between raised HER2 and tumour marker (CA15.3 and CEA) expression observed in the present study (Table 3), is that these changes may simply be a reflection of tumour burden, in which a certain critical level of cells expressing HER2 is necessary for its detection in serum [18,19]. Furthermore, these changes may be a reflection of genetic instability within the primary tumour. Studies have shown that breast cancer cells that survive adjuvant treatment may undergo upregulation or downregulation of some biological markers [20]. Specifically with regards to upregulation of HER2, Slichenmyer and Fry [21] and Tanner and coworkers [22] demonstrated this to occur in breast cancer relapsing after adjuvant hormone therapy.

In this study we were able to obtain paired tissue samples from eight patients and, in all, no change in tissue HER2 expression was observed. Of these, HER2 serum expression at metastasis was normal in seven, although in one (which exhibited tissue over-expression at primary) a change in serum expression was observed. This may suggest an association between tissue expression at primary and serum expression at metastases, although statistical analysis on such a small group is not valid. Similar findings have been reported by others in a larger case series [23]. However, the literature reports conflicting findings on the expression of HER2 in primary and metastastic tissue; some studies have identified no difference, whereas others have shown a difference in up to 26% of cases [18,22,24,25]. Most of these studies are small case series, and understandably so because metastatic tissue for analysis in breast cancer is not readily available. Indeed, this is the only study, as far as we are aware, whereby an attempt is made to analyze both serum and tissue of both the primary and metastases in the same patient.

Serum EGFR levels in our group of breast cancer patients were significantly lower when compared with those in healthy control individuals. Although this observation is supported by others [6], it is noteworthy that in our healthy control group 20 (40.8%) had raised serum EGFR levels. We would have expected 95% of this group to fall within the reference range, and it must be assumed that this is due to differences in our control group and the control group evaluated by Bayer to produce the reference range. A 95% reference range is based on a normal distribution of the data, but serum EGFR (as with tumour markers in general) was not normally distributed in our control group. Also, our control group included a combination of women who had normal mammograms as part of the National Health Service Breast Cancer Screeening Program and those who had attended a symptomatic breast clinic but had a normal assessment. It may therefore be that benign breast diseases effect serum EGFR levels. Finally, differences in menopausal status between these two groups may have an influence (see below). However, we have not evaluated this specifically.

In addition to these findings, a significant decrease in serum expression of EGFR was observed between the diagnoses of PBC and of metastatic disease. This phenomenon, whereby a marker decreases with progression of disease, is not commonly seen and suggests that this marker may provide different information to that from CA15.3, CEA and HER2. Several hypotheses for this observation have been proposed. One is that the proteolytic cleavage of the extracellular domain of the EGFR receptor decreases as normal cells progress to cancer cells. It has also been suggested that that this may be related to the association between circulating serum oestrogen and serum EGFR levels shown in several studies. In a study conducted by Baron and colleagues postmenopausal women had significantly lower sEGFR levels than did premenopausal women [13]. Also, in another study conducted by Lafky and coworkers [26] in metastatic breast cancer patients treated with an aromatase inhibitor (letrozole), post-treatment serum EGFR levels (measured at 1 and 3 months) were significantly lower than pretreatment levels.

In our study, patients who were ER positive and on hormone treatment with tamoxifen at the time of metastatic diagnosis were significantly less likely to exhibit a reduction in serum EGFR levels when compared with patients who were not receiving treatment (Table 2). The fact that tamoxifen does not appear to decrease serum oestrogen levels may in part explain this observation [27], as discussed above. In addition, there is an inherent selection bias in this group, because patients not on treatment probably had a longer disease-free interval and were therefore potentially more likely to have gone from being premenopausal at primary diagnosis to postmenopausal at metastasis. However, this was not assessed specifically in our study. We were able to evaluate five paired tissue samples for EGFR expression, and in this small group of samples only one ehibited a definitive change from no expression to positive expression (on membrane expression analysis). Although only a very small number of samples was included in this analysis, we did not observed the same trend toward a reduction in expression as observed in serum.

Contradictory findings have been reported in the literature with regard to the prognostic information provided by serum EGFR assessments, because some have shown low levels to be of importance whereas others suggest the contrary [6,23,26]. However, until it can be established exactly what serum EGFR is measuring, it is not as useful a clinical tool as serum HER2 [3].

ErbB receptors exist as plasma membrane monomers. After binding of the appropriate soluble extracellular ligand, the monomeric receptors dimerize and become functionally active. They can form homodimers or heterodimers with other members of the ErbB family, and recent studies have shown that there is considerable crosstalk (cross-phosphorylation) between the EGFR and HER2 receptors [28]. It has been postulated that this crosstalk could explain trastuzumab resistance in some HER2-positive patients and that by preventing this cross-talk (with EGFR inhibition) the efficacy of trastuzumab may be enhanced [5]. In our study, EGFR levels were elevated in 21% of metastatic breast cancer patients, and in 12% serum levels of both EGFR and HER2 were elevated. This group of patients is of interest because *in vitro *studies suggest that they may gain particular benefit from the combination of anti-EGFR and anti-HER2 treatment [29].

Addition of assessment of serum HER2 and serum EGFR to CEA and CA15.3 into a 'tumour marker profile' significantly increases the chances that one of these markers will be raised (52% to 78%; Table 3). This is in agreement with other studies and may be helpful in the early diagnosis of tumour recurrence or metastases or to assess response to treatment [30,31]. At the present time, however, the use of tumor markers for this purpose in breast cancer patients is not recommended, according to international guidelines.

## Conclusion

This study shows that, in a number of breast cancer patients, serum levels of the oncogenes HER2 and EGFR change significantly between primary diagnosis and the onset of metastatic disease, albeit in opposite directions. Decisions on use of targeted therapies in the metastatic setting are often based on the oncogene expression of the primary tumour. More controversial is whether elevated serum HER2 and/or serum EGFR levels might be used to select patients for targeted therapies, even if the primary tumour tissue did not over-express these oncogenes. This is an important question that almost certainly will require a multi-institutional study to address.

## Abbreviations

CA = cancer antigen; CEA = carcinoembryonic antigen; EGFR = epidermal growth factor receptor; ER = oestrogen receptor; HER = human epidermal growth factor receptor; PBC = primary breast cancer.

## Competing interests

The authors declare that they have no competing interests.

## Authors' contributions

KSA was responsible for acquisition of data, with contributions from AA, CA, AH and CC. KSA drafted the manuscript. IOE, KLC and JFRR revised the manuscript for important intellectual content, and JFRR gave final approval of the version to be published.
